# A novel chemical inhibitor suppresses breast cancer cell growth and metastasis through inhibiting HPIP oncoprotein

**DOI:** 10.1038/s41420-021-00580-3

**Published:** 2021-07-29

**Authors:** Pengyun Li, Shengjie Cao, Yubing Huang, Yanan Zhang, Jie Liu, Xu Cai, Lulu Zhou, Jianbin Li, Zefei Jiang, Lihua Ding, Zhibing Zheng, Song Li, Qinong Ye

**Affiliations:** 1 Department of Medical Molecular Biology, Beijing Institute of Biotechnology, Beijing, 100850 China; 2 Laboratory of Computer-Aided Drug Design & Discovery, Beijing Institute of Pharmacology and Toxicology, Beijing, 100850 China; 3 Fifth Medical Center of PLA General Hospital, Beijing, 100071 China

**Keywords:** High-throughput screening, Breast cancer

## Abstract

Increasing evidence suggests the pivotal role of hematopoietic pre-B-cell leukemia transcription factor (PBX)-interacting protein (HPIP/PBXIP1) in cancer development and progression, indicating that HPIP inhibition may be a promising target for cancer therapy. Here, we screened compounds inhibiting breast cancer cell proliferation with HPIP fused with green fluorescent protein as a reporter. A novel agent named TXX-1-10 derived from rimonabant, an antagonist of cannabinoid receptor 1 with anticancer effects, has been discovered to reduce HPIP expression and has greater inhibitory effects on breast cancer cell growth and metastasis in vitro and in vivo than rimonabant. TXX-1-10 regulates HPIP downstream targets, including several important kinases involved in cancer development and progression (e.g., AKT, ERK1/2, and FAK) as well as cell cycle-, apoptosis-, migration-, and epithelial-to-mesenchymal transition (EMT)-related genes. Consistent with the results of anticancer effects, genome-wide RNA sequencing indicated that TXX-1-10 has more significant effects on regulation of the expression of genes related to DNA replication, cell cycle, apoptosis, cell adhesion, cell migration, and invasion than rimonabant. In addition, TXX-1-10 significantly regulated genes associated with the cell growth and extracellular matrix organization, many of which were shown to be regulated by HPIP. Moreover, compared with rimonabant, TXX-1-10 greatly reduces blood-brain barrier penetrability to avoid adverse central depressive effects. These findings suggest that HPIP inhibition may be a useful strategy for cancer treatment and TXX-1-10 is a promising candidate drug for cancer therapy.

## Introduction

As one of the most common heterogeneous diseases among women, breast cancer highly displays diversification in terms of its presentation, disease progression, pathological characteristics, and clinical response [1, 2]. Although great progress has been made in clarifying molecular features and pathogenesis underlying breast tumorigenesis, and multiple therapeutic strategies have been employed in individual treatment, some types of breast cancer patients develop with aggressive characteristics and have a poor prognosis [3, 4]. Therefore it is necessary to investigate the factors regulating cancer progression and develop novel molecules for cancer treatment.

The PI3K/Akt/mTOR and RAS-RAF-MEK-ERK signaling pathways are hyperactivated in a high percentage of tumors, including epithelial ovarian, glioblastoma, breast, lung, and renal cancers [5–7]. Increasing studies have been focused on the development of small-molecule inhibitors targeting components of PI3K/Akt/mTOR and RAS-RAF-MEK-ERK signaling as cancer therapeutics [8]. Recently, AKT inhibition using small-molecule inhibitors, such as AZD5363 and GSK690693 have been studied in preclinical setup and evaluated in many clinical trials to block human tumors [9]. ERK inhibitors such as Ulixertinib were used for the treatment of various advanced/metastatic solid tumors [10]. However, clinical efficacy using single molecules directly inhibiting Akt or ERK signaling was shown to have a limited pharmaceutical effect on cancer due to the induction of resistance [9, 11]. Moreover, several therapies have been discontinued because of the development of side effects, such as rash, hyperglycemia, and hemolytic toxicity after intravenous injection [6], implying the need for more innovative approaches to achieve inhibition of Akt or ERK signaling for cancer treatment with minimal side effects.

Hematopoietic PBX-interacting protein (HPIP), a corepressor for the transcription factor PBX, is involved in organogenesis and tumorigenesis [12, 13]. We and others have previously reported the critical role of HPIP for the progression in various human cancer types, including infiltrative ductal carcinoma [14], liver cancer [15], gastric cancer [16], and colorectal carcinoma [17]. HPIP modulates hepatocellular carcinoma cell growth, invasion, epithelial-mesenchymal transition (EMT), and metastasis through regulating the AKT, ERK, and mTOR signaling [15]. HPIP is also an activator of focal adhesion kinase (FAK) to regulate cell adhesion and migration [14]. A very recent study shows that HPIP promotes the G2/M transition of the cell cycle by stabilizing cyclin B1 [18]. Due to the importance of HPIP in cancer, we screened chemical compounds inhibiting breast cancer cell proliferation with HPIP fused with green fluorescent protein as a reporter. A novel small molecule named TXX-1-10 derived from rimonabant, an antagonist for the cannabinoid receptor CB1 was identified to reduce HPIP expression together with its downstream targets and inhibit tumor growth and metastasis with great efficacy than rimonabant. In addition, compared with rimonabant, TXX-1-10 considerably reduces blood-brain barrier penetrability, thereby avoiding adverse central depressive effects.

## Results

### Identification of the small chemical molecule TXX-1-10 that inhibits HPIP expression by cell-based screening

**Fig. 1:**
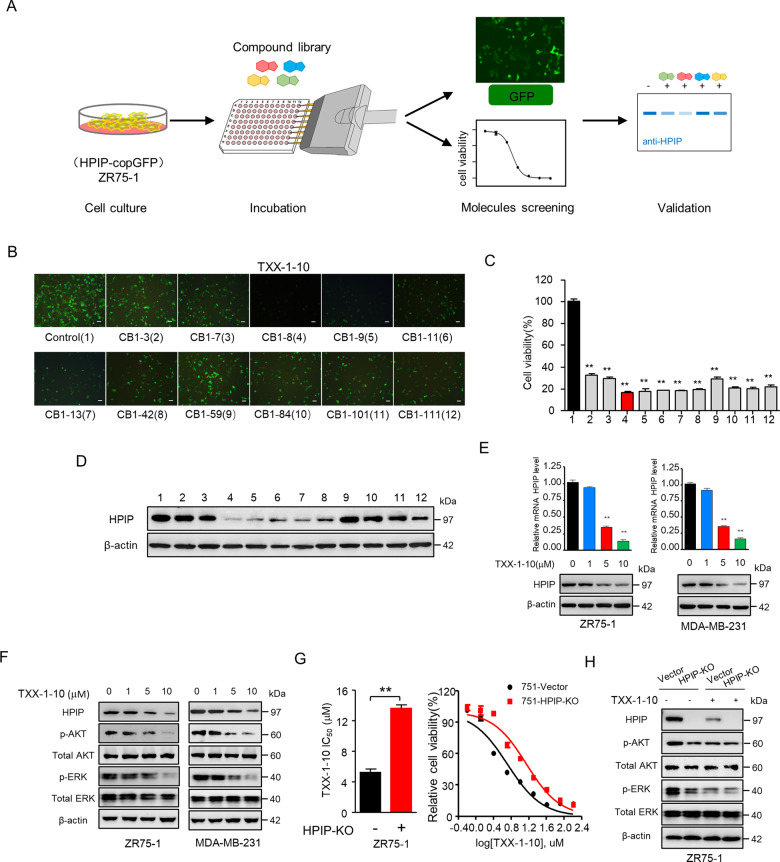
Identification of the small chemical molecule TXX-1-10 inhibiting HPIP expression by cell-based screening. **A** Schematic representation of the protocol used to screen small molecules targeting HPIP. **B** Representative images of ZR75-1 cells stably expressing HPIP-copGFP treated with the indicated rimonabant derivatives for 48 h (10 μM). Scale bars:100 μm. **C** The antiproliferation function of the indicated rimonabant derivatives as in **B** of three independent experiments in ZR75-1 cells. **D** Western blot analysis of ZR75-1 cells treated with the indicated rimonabant derivatives as in **B** for 24 h. **E** qRT-PCR and Western blot analysis of relative HPIP expression in ZR75-1 and MDA-MB-231 cells treated with the indicated concentrations of TXX-1-10 for 24 h. **F** Western blot analysis of ZR75-1 and MDA-MB-231 cells with increasing concentrations of TXX-1-10 for 24 h. **G** Viability curves of wild type or HPIP knockout (KO) ZR75-1 cells treated with the indicated concentrations of TXX-1-10 for 48 h. TXX-1-10 IC_50_ was assessed after 48 h. **H** Western blot analysis of ZR75-1 with TXX-1-10 (10 μM) treatment for 24 h or HPIP knockout. Data shown are mean ± SD of triplicate measurements that have been repeated three times with similar results. Statistical significance was assessed by two-tailed Student’s *t* test. *p < 0.05, **p < 0.01 versus the corresponding control.

Since the crucial role of HPIP in cancer development and progression, we developed an image-based screening model with HPIP-copGFP (green fluorescent protein)-overexpressing ZR75-1 cells to screen small chemical molecules that decrease both fluorescence signal intensity and cell viability. The screened molecules were further quantitatively validated by Western blot (WB) with anti-HPIP (Fig. 1A). Interestingly, the primary screen based on a library of over 600 compounds revealed that molecules derived from rimonabant significantly reduced both fluorescence intensity and cell viability (Supplementary Fig. 1A). Among these compounds, TXX-1-10 (CB1-8) exhibited the most significant effect on reducing cell viability, fluorescence intensity, and HPIP protein expression (Fig. 1B–D and Supplementary Fig. 1B, C). Quantitative reverse-transcription PCR (RT-qPCR) and western blot analysis indicated that TXX-1-10-reduced HPIP mRNA and protein levels in a dose-dependent manner (Fig. 1E). Consistent with the results showing that HPIP-activated AKT and ERK1/2 [15], TXX-1-10-repressed AKT and ERK1/2 phosphorylation in a dose-dependent manner (Fig. 1F). In addition, HPIP knockout decreased the sensitivity of ZR75-1 cells to TXX-1-10 as well as the effect of TXX-1-10 on reducing AKT and ERK1/2 phosphorylation (Fig. 1G, H). Taken together, these data demonstrate that TXX-1-10 reduces HPIP expression and exerts antiproliferation effect in a HPIP-dependent manner.

### TXX-1-10 inhibits cancer cell proliferation

**Fig. 2:**
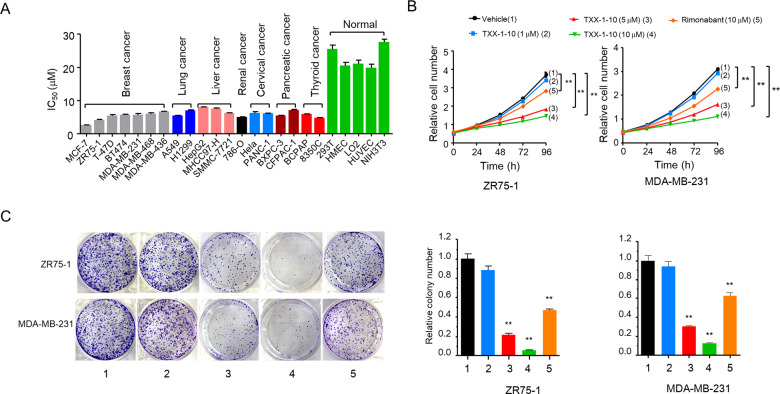
TXX-1-10 inhibits cancer cell proliferation. **A** IC_50_ values of TXX-1-10 in 24 cell lines were determined by the CCK8 assay. **B** The proliferation curve of ZR75-1 and MDA-MB-231 cells treated with indicated concentrations TXX-1-10, rimonabant or vehicle control (DMSO). **C** Colony formation assays for ZR75-1 and MDA-MB-231 cells treated as in **B**. Histograms show the colony number (right panel). Data shown are mean ± SD of triplicate measurements that have been repeated three times with similar results. Statistical significance was assessed by two-tailed Student’s *t* test. *p < 0.05, **p < 0.01 versus the corresponding control.

Next, we investigated the effect of TXX-1-10 on cancer cell viability in 19 cancer cell lines and five normal cell lines. The IC_50_ values in various cancer cell lines varied from 2.2 to 7.9 µM, indicating that TXX-1-10 decreases cancer cell viability without cell-type specificity (Fig. 2A and Supplementary Fig. 1D). The IC_50_ values in the normal cell lines ranged from 19.7 to 27.5 µM, suggesting that cancer cells are more sensitive to TXX-1-10 than normal cells. We next explored the level of HPIP in the panel of breast cancer cell lines, which was significantly reduced with TXX-1-10 treatment. In contrast, the expression of HPIP in the normal counterpart HMEC cells was not decreased with TXX-1-10 treatment (Supplementary Fig. 1E). In addition, cell proliferation assay and colony formation assay showed that TXX-1-10 exhibited a more effective inhibition compared with rimonabant in MDA-MB-231 and ZR-751 cells (Fig. 2B, C).

Since rimonabant was withdrawn from the market because of its neuropsychiatric adverse events [19], we detected the blood-brain barrier (BBB) permeability of rimonabant and TXX-1-10. Compared with rimonabant, TXX-1-10 exhibited a dramatic reduction of logBB, S + logP and S + logD predicted by ADMET Predictor 8.1 program (Supplementary Fig. 3). TXX-1-10 showed a 15-fold decrease in BBB permeability than rimonabant in rat brain uptake assay, implying that TXX-1-10 possessed little psychiatric side effects compared with rimonabant (Supplementary Fig. 4). Taken together, these data demonstrated that TXX-1-10 reduces cancer cell proliferation with reduced BBB permeability.

### TXX-1-10 induces G1 cell cycle arrest and apoptosis in breast cancer cells

**Fig. 3:**
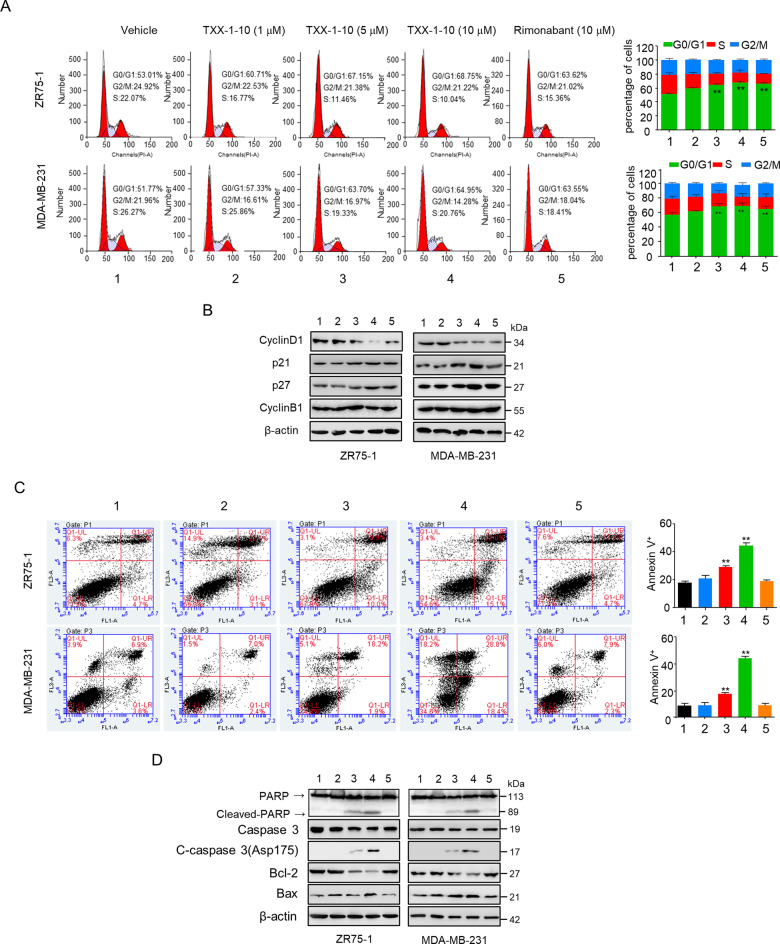
TXX-1-10 induces G1/S cell cycle arrest and apoptosis in breast cancer cells. **A** The cell cycle distributions of ZR75-1 and MDA-MB-231 cells treated with TXX-1-10, Rimonabant, or vehicle for 48 h. The cell cycle was evaluated by flow cytometry after staining with propidium iodide (PI) for 30 min at 37 °C in dark. Histograms show the cell cycle distributions of ZR75-1 and MDA-MB-231 cells (right panel). **B** Representative immunoblot assay of ZR75-1 and MDA-MB-231 cells treated as in A with the indicated antibodies. **C** Representative images of flow cytometry analysis of apoptosis in ZR75-1 and MDA-MB-231 cells treated as in **A**. Statistical analysis of apoptosis rates was shown in the right panel. **D** Representative immunoblot assay of ZR75-1 and MDA-MB-231 cells from **C** with the indicated antibodies. Statistical significance was assessed by two-tailed Student’s *t* test. *p < 0.05, **p < 0.01 versus the corresponding control.

Since anticancer drugs eliminate tumor cells predominantly by triggering apoptosis and inducing cell cycle arrest [20, 21], we next examined the effect of TXX-1-10 on breast cancer cell cycle and apoptosis. Compared with the vehicle, TXX-1-10 increased the proportion of cells in G0/G1 phase and decreased the proportion of cells in S-phase and G2/M-phase of both ZR-751 and MDA-MB-231 in a dose-dependent manner, suggesting that TXX-1-10 induces G1 cell cycle arrest in breast cancer cells (Fig. 3A). To elucidate the mechanism by which TXX-1-10 induces G1 cell cycle arrest, we examined the expression of several important cell cycle-related proteins. Consistent with the results of TXX-1-10-induced G1 cell cycle arrest, TXX-1-10 significantly reduced the expression of the G1/S phase marker cyclin D1 and increased the levels of p27 and p21, two major regulators of G1/S phase transition. However, TXX-1-10 did not alter cyclin B1 expression, which is predominantly expressed during G2/M phase (Fig. 3B). Next, we determined whether TXX-1-10 regulates apoptosis of breast cancer cells. Compared with the control, TXX-1-10 significantly induced the apoptosis of ZR-751 (from 15.8 to 42.1%) and MDA-MB-231 (from 10.5 to 47.2%) cells (Fig. 3C). In Consistent with the results of TXX-1-10-induced apoptosis, TXX-1-10 inhibited the expression of the antiapoptotic protein Bcl-2, and promoted the expression of BAX, cleaved PARP, and cleaved-caspase 3 that are the apoptotic proteins in a dose-dependent manner (Fig. 3D). However, rimonabant did not induce apoptosis or alter the expression of these apoptosis-related proteins (Fig. 3C, D). Taken together, these data demonstrated that TXX-1-10 induce G1 cell cycle arrest and apoptosis in breast cancer cells.

### TXX-1-10 inhibits breast cancer cell migration and invasion in vitro

**Fig. 4:**
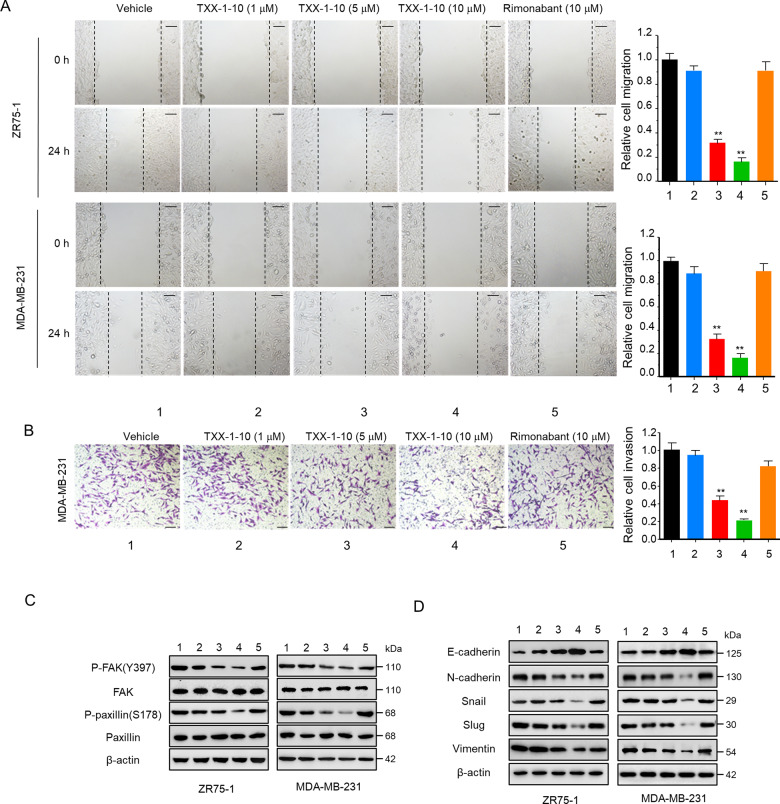
TXX-1-10 inhibits breast cancer cell migration and invasion. **A** Wound-healing assay of ZR75-1 and MDA-MB-231 cells treated with TXX-1-10, rimonabant or vehicle control (DMSO) following mitomycin C (1 μM) treatment at the indicated time. Histograms show the relative cell migration (right panel). Scale bar: 100 μm. **B** Transwell assay of MDA-MB-231 cells treated as in **A**. Invasive cells were fixed and stained with crystal violet. Histograms show the relative cell invasion (bottom). **C** Western blot assay of ZR75-1 and MDA-MB-231 cells treated with increasing concentrations of TXX-1-10 for 24 h with the indicated antibodies. **D** Western blot assay of ZR75-1 and MDA-MB-231 cells treated with increasing concentrations of TXX-1-10 for 24 h with the indicated antibodies. Data shown are mean ± SD of triplicate measurements that have been repeated three times with similar results. Statistical significance was assessed by two-tailed Student’s *t* test. *p < 0.05, **p < 0.01 versus the corresponding control.

Tumor metastasis is the most common leading cause of breast cancer-related mortality and remains the greatest challenge in clinical cancer management, so it is urgent to develop novel potential candidates to cope with metastatic progression [4, 22, 23]. Next, we examined the effect of TXX-1-10 on migration and invasive capacity in breast cancer cells. Wound-healing assay and matrigel invasion assay showed that TXX-1-10 specifically inhibited the migration and invasion of ZR-751 and MDA-MB-231 in a dose-dependent manner (Fig. 4A, B). In contrast, rimonabant had little effect on the migration and invasion of breast cancer cells.

Since HPIP has been reported to be an activator of focal adhesion kinase (FAK) to regulate cell adhesion and migration [14], and phosphorylation of FAK (Tyr397) and Paxillin (Ser178) promote cancer cell migration and invasion [24], we investigated whether TXX-1-10 regulates FAK and Paxillin [14]. Indeed, TXX-1-10 inhibited the phosphorylation of FAK (Tyr397) and Paxillin (Ser178) in a dose-dependent manner, and rimonabant had less inhibitory effect compared with TXX-1-10 (Fig. 4C). As migration defects are often associated with markers of EMT [23], and HPIP has also been shown to promote renal cell carcinoma migration and invasion with increased EMT [25]. Next, we found that TXX-1-10 significantly increased the expression of the epithelial marker E-cadherin. In comparison, the expression of mesenchymal marker N-cadherin, Vimentin, snail, and slug was significantly reduced with TXX-1-10 treatment (Fig. 4D). ALDH1-high cells are considered as breast cancer stem cells (CSCs). Neither TXX-1-10 nor rimonabant showed the effect on ZR75-1 and MDA-MB-231 cell stemness (Supplementary Fig. 5A, B). Taken together, these data indicated that TXX-1-10 inhibits breast cancer cell migration and invasion.

### RNA-Seq reveals TXX-1-10 regulating expression of genes related to cell growth and metastasis

**Fig. 5:**
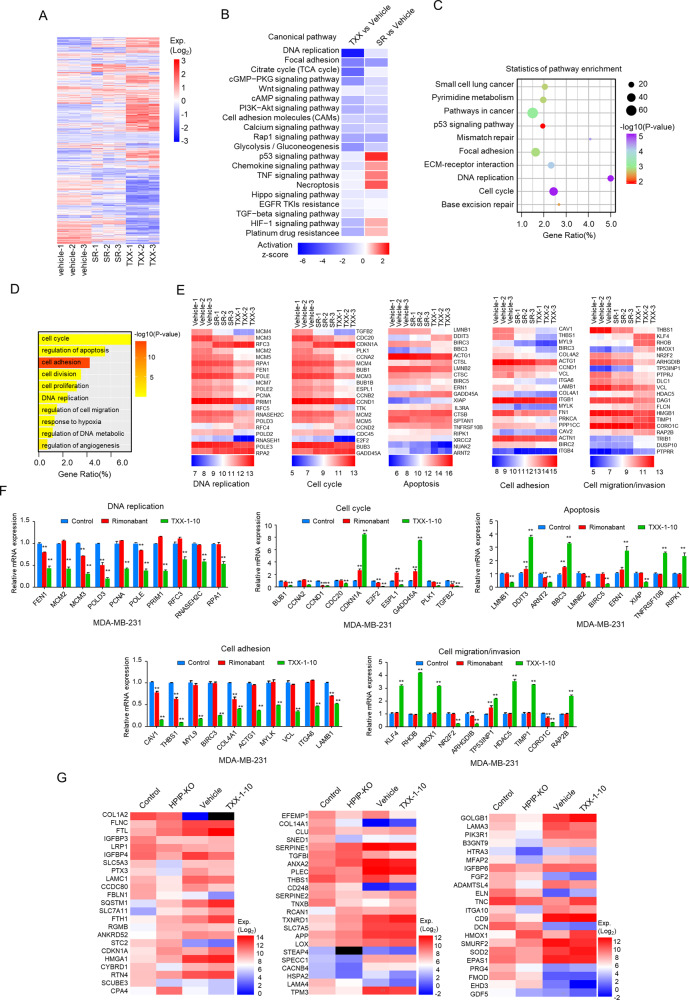
RNA-seq reveals TXX-1-10 regulates genes related to cell proliferation and invasion. **A** Heatmap of transcriptional profile denoting unbiased clustering of MDA-MB-231 cells treated with rimonabant (SR), TXX-1-10(TXX) or vehicle control (DMSO) (*n* = 3). Total RNA was analyzed by high-throughput whole transcriptome sequencing (RNA-seq). Significant differential expression is defined as an absolute log2 (fold change) ≥1 and *q* < 0.05. **B** Comparative pathway analysis of mRNA expression in MDA-MB-231 cells treated with TXX-1-10 (TXX) and rimonabant (SR). **C** KEGG pathway analysis of targets regulated by TXX-1-10 (The top 10 most significantly affected pathways are shown). **D** Gene ontology (GO) functional clustering of genes that were downregulated in MDA-MB-231 cells treated with TXX-1-10. **E** Heatmap of significantly regulated genes by TXX-1-10 and rimonabant correlated with DNA replication, cell cycle, apoptosis, cell adhesion, and cell migration/invasion/metastasis (*n* = 3, the top 20 most significantly affected genes are shown). **F** qRT-PCR analysis of the indicated gene expression associated with DNA replication, cell cycle, apoptosis, cell adhesion, and cell migration/invasion/metastasis in MDA-MB-231 cells treated with TXX-1-10 and rimonabant. **G** Heatmap of significantly regulated genes by TXX-1-10 and HPIP-KO associated with the cell growth and ECM organization. Data shown are mean ± SD of triplicate measurements with similar results. Statistical significance was assessed by two-tailed Student’s *t* test. *p < 0.05, **p < 0.01 versus the corresponding control.

Given TXX-1-10 as a potent tumor-suppressive agent, we investigated the impact of TXX-1-10 on global gene expression using RNA sequencing (RNA-seq) (Fig. 5A and Supplementary Fig. 7A, B). RNA-seq assay demonstrated an extensive overlap of genes commonly regulated by TXX-1-10 and rimonabant. The expression of 78.6% of the genes changed in rimonabant treatment was recapitulated in the TXX-1-10-treated cells (Supplementary Fig. 7C).

Analysis of common regulated genes by TXX-1-10 and rimonabant showed that TXX-1-10 and rimonabant generally exerted inhibitory effects on diverse tumor-associated biological processes, such as DNA replication, focal adhesion, and cancer-related pathways including cGMP-PKG signaling pathway, PI3K-Akt signaling pathway, and HIF-1 signaling pathway (Fig. 5B). TXX-1-10 exhibited a more effective inhibition of these processes compared with rimonabant. KEGG analysis of TXX-1-10-regulated 3235 (10.72%) genes demonstrated that TXX-1-10-modulated signaling pathways, such as DNA replication, cell cycle, and focal adhesion, which were essential for cancer cell proliferation and/or metastasis (Fig. 5C). GO enrichment analysis showed that TXX-1-10-regulated genes also implicated in the cancer cell biological processes, such as DNA replication (GO: 0006260), cell cycle (GO: 0007049), and apoptosis (GO: 0042981) (Fig. 5D). Importantly, some of these transcripts regulated by TXX-1-10 and rimonabant were further validated by qRT-PCR, and TXX-1-10 exhibited a greater impact on the expression of genes involved in DNA replication, cell cycle, apoptosis, cell adhesion, and cell migration, invasion and metastasis than rimonabant (Fig. 5E, F and Supplementary Fig. 7D–H). It has been reported that genes related to skeletal system development, cell growth, and extracellular matrix organization related to cell migration, invasion, and metastasis were regulated in HPIP-deficient transcriptome [26]. We also found that the expression of those 68 genes were also changed in the TXX-1-10-treated cells (Fig. 5G). Collectively, these findings demonstrated TXX-1-10 modulates the expression of many genes implicated in cancer cell growth and metastasis, many of which are regulated by HPIP.

### TXX-1-10 suppresses breast tumor growth and metastasis in vivo

**Fig. 6:**
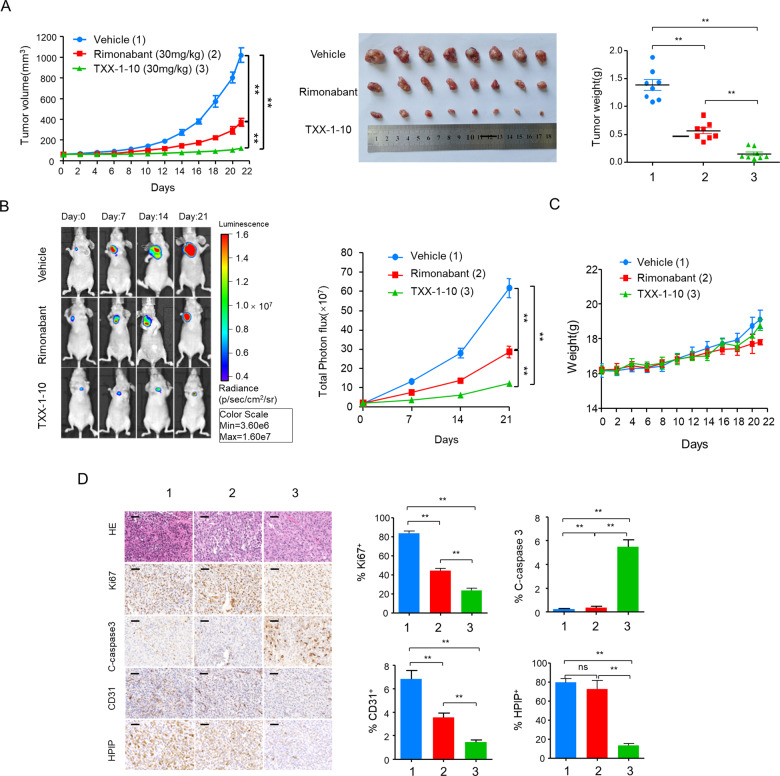
TXX-1-10 represses breast tumor growth in vivo. **A** Bioluminescent MDA-MB-231 cells were subcutaneous transplanted into BALB/c mice. Mice were treated with vehicle control, rimonabant (30 mg/kg) or TXX-1-10 (30 mg/kg) 5 days after xenograft as indicated and tumor size was monitored every other day. Photograph of tumors and tumor weight of animals administrated with TXX-1-10, rimonabant or vehicle control are shown (*n* = 8 per group). **B** Representative bioluminescent images of animals were taken at the day 0, 7th-, 14th-, and 21st-day postbioluminescent MDA-MB-231 xenografts (left panel). Quantification of tumor cells in mice body was performed with bioluminescence analysis (right panel) (*n* = 8 per group). **C** Body weight of BALB/c mice treated with vehicle control, rimonabant (30 mg/kg) or TXX-1-10 (30 mg/kg) as indicated are shown (*n* = 8 per group). **D** Representative images of H&E, Ki67, cleaved-caspase 3, HPIP, and CD31 immunohistochemical (IHC) staining in harvested tumors from each group are shown (left panel). Histograms show the quantification of Ki67^+^, cleaved-caspase 3^+^ cells, CD31^+^ cells, and HPIP ^+^ cells (right panel). Scale bar: 50 μm. Data shown are mean ± SD of triplicate measurements that have been repeated three times with similar results. *p* values were determined by two-tailed Student’s *t* test (*p < 0.05, **p < 0.01). Statistical significance was assessed by two-tailed Student’s *t* test. (ns not significant; *p < 0.05, **p < 0.01). **p < 0.01 on the final day **A**, **B**.

**Fig. 7:**
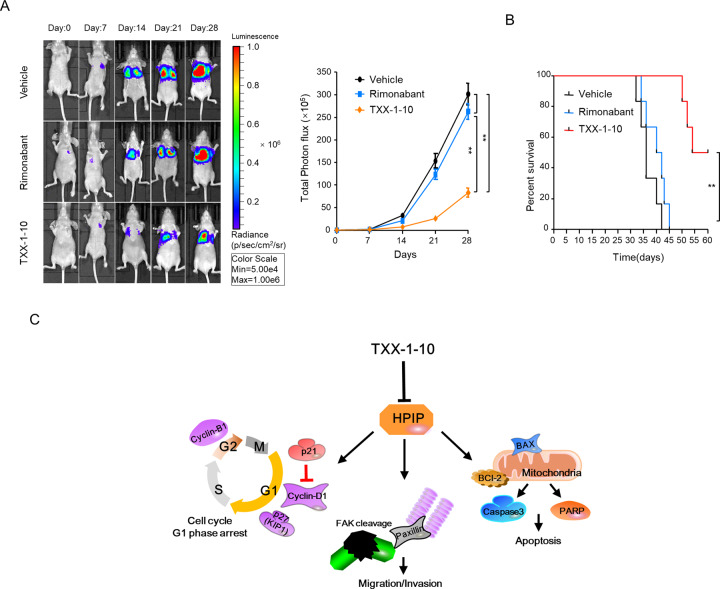
TXX-1-10 represses breast tumor metastasis in vivo. **A** Bioluminescent MDA-MB-231 cells were tail vein injected into BALB/c mice. Mice were treated with vehicle control, rimonabant (30 mg/kg) or TXX-1-10 (30 mg/kg) as indicated. Representative images were taken at the 0, 7th, 14th, and 21st day, respectively (left panel). Histograms show the quantification of metastatic cells in the whole body determined by bioluminescence analysis (right panel) (*n* = 6 per group). **B** Survival rates of nude mice treated as in **A** were shown (*n* = 6 per group). **C** Proposed model for TXX-1-10 inhibition of breast cancer growth and metastasis. TXX-1-10 decreases HPIP expression, leading to the regulation of inducing cell cycle arrest, apoptosis and inactivation of the metastasis-associated gene, causing growth and metastasis inhibition of breast cancer. *p* values were determined by two-tailed Student’s *t* test (*p < 0.05, **p < 0.01). Statistical significance was assessed by two-tailed Student’s *t* test. (ns not significant; *p < 0.05, **p < 0.01). **p < 0.01 on the final day **A, B**.

Since the TXX-1-10 exhibits a stronger inhibitory effect than rimonabant on breast cancer cell proliferation and motility in vitro, we examined the effect of TXX-1-10 and rimonabant on breast cancer growth and metastasis in vivo. Consistent with the results in vitro, the TXX-1-10 showed a stronger inhibitory effect on breast tumor growth than rimonabant without affecting the body weight of mice (Fig. 6A–C). Compared to the rimonabant groups, the TXX-1-10 groups showed a more significant inhibitory effect on the amount of Ki67 (proliferation marker)-, CD31 (endothelial cell marker)-, and HPIP-positive tumor cells, and a more significant stimulatory effect on the amount of cleaved caspase-3 (apoptosis marker)-positive tumor cells (Fig. 6D), in agreement with the results of the effects of TXX-1-10 and rimonabant on breast tumor growth in vivo. Furthermore, the lung metastasis of the TXX-1-10 treatment group was significantly decreased compared with that of the rimonabant group (Fig. 7A), and the survival rates of the TXX-1-10 (P < 0.05) groups were higher than those of the rimonabant groups (Fig. 7B). Taken together, these data suggested that the TXX-1-10 inhibits breast tumor growth and lung metastasis, and TXX-1-10 shows a more potent anticancer effect than rimonabant.

## Discussion

In the present study, we developed a novel agent named TXX-1-10, a derivative of rimonabant, which significantly reduces HPIP expression and regulates HPIP downstream targets, such as AKT, ERK1/2, and FAK as well as cell cycle-, apoptosis- and migration/invasion-related genes (Fig. 7C). The TXX-1-10 has a more effective inhibition on breast cancer cell proliferation, migration, invasion, and metastasis in vitro and in vivo than rimonabant. Genome-wide RNA sequencing indicated TXX-1-10 has more significant effects on the regulation of the expression of genes involved in cancer cell growth and metastasis than rimonabant. Furthermore, compared with rimonabant, the blood-brain barrier permeability of TXX-1-10 is decreased to avoid psychotropic side effects. These findings suggest that the TXX-1-10 can be a candidate drug for inhibition of cancer growth and metastasis.

Since HPIP modulates cancer cell growth, invasion, epithelial-mesenchymal transition (EMT) and metastasis through regulating various genes, including AKT, ERK, FAK, and mTOR signaling [13–15, 25, 27]. Herein, we found TXX-1-10-reduced AKT and ERK1/2 phosphorylation in a dose-dependent manner. Moreover, knockout of HPIP alleviated the effect of TXX-1-10 on AKT and ERK inhibition, demonstrating the role of HPIP in TXX-1-10-regulated AKT and ERK signaling. It has also been reported that HPIP interacts with ERα and increasing ERα target genes expression, including pS2 and cathepsin D through activation of MAPK and AKT [13]. In this paper, we also explored whether TXX-1-10 treatment affect ERα expression, HPIP-ERα cooperation as well as the modulation of ERα target genes. TXX-1-10 treatment showed no effect on HPIP-ERα interaction (Supplementary Fig. 6A); however, TXX-1-10-reduced the expression of pS2 and cathepsin D, two ERα target genes (Supplementary Fig. 6B), which may be modulated by inhibition of MAPK and AKT with TXX-1-10 treatment (Supplementary Fig. 5D). In addition, overexpression of HPIP reversed the inhibitory effect of TXX-1-10 on pS2 and cathepsin D.

In the last few decades, an increasing number of studies have revealed the aberrant expression of CB1 receptor in numerous types of tumors, which is related to cancer prognosis and disease outcome [28, 29]. Several authors have demonstrated that CB1 antagonist rimonabant exerts an anticancer activity in various cancers, including breast cancer [30] and colon cancer [31, 32]. Here, we have also shown that rimonabant suppresses the breast cancer cell proliferation and tumor growth both in vitro and in vivo. However, it was found that rimonabant had neuropsychiatric adverse events, such as alteration of emotional behaviors and cognitive function, which led to its withdrawal from the market [19]. In this study, we developed TXX-1-10, a derivative of rimonabant with a more potent antitumor capacity in inhibiting tumor growth and metastasis than rimonabant both in vitro and in vivo. The antiproliferative effects of TXX-1-10 are accompanied by substantial apoptotic processes along with increased caspase-3-mediated PARP cleavage as well as the reduced ratio of Bcl-2/Bax and subsequent G1 cell cycle arrest. Although rimonabant also induced G1 cell cycle arrest, no effects on cell apoptosis and apoptotic-related proteins were observed. In addition, TXX-1-10 inhibits cancer metastasis via decreasing the phosphorylation and catalytic activity of proteins related to the FAK signal pathway, whereas rimonabant showed a little suppressive effect of cancer metastasis as well as related proteins. Furthermore, RNA-sequencing analysis also reveals that TXX-1-10 generally leads to a more significant suppression or stimulation of genes than rimonabant involved in tumor-associated biological processes.

Given the side effects of rimonabant, we detected the BBB permeability of TXX-1-10. The results indicate that the latter would not exert unwanted psychotropic effects as the three-position of the pyrazole ring of rimonabant is structurally modified with a higher hydrophilic quaternary ammonium salt group. Indeed, the physicochemical properties predicted by the ADMET predictor 8.1 program and brain uptake experiment in rats both confirmed a much lower logBB, S + logP and S + logD of TXX-1-10 than those of rimonabant. Furthermore, TXX-1-10 was well tolerated in animal studies and showed no side effects on the body weight of mice after 20 days of treatment, indicating that it has fewer side effects. The physicochemical property prediction also suggests the satisfactory physicochemical properties and low toxicological effects of TXX-1-10. However, the toxicity and physiochemical properties of TXX-1-10 in vivo require more detailed investigations. Future studies are also warranted to evaluate the optimized dose and treatment conditions of such an agent as a clinical candidate drug for cancer treatment.

Except for defining tumor-suppressive properties of TXX-1-10, it is still necessary to fully understand how TXX-1-10 regulates gene expression and signaling pathways. To investigate the molecular mechanisms underlying the function of TXX-1-10, we performed RNA-Seq analysis to search for TXX-1-10-mediated pathways. Gene ontology and KEGG enrichment analysis revealed that TXX-1-10 modulates genes associated with numerous cellular processes, including DNA replication, cell cycle, apoptosis, focal adhesion, angiogenesis, and energy metabolism. These processes contribute to cancer development and progression and belong to certain hallmarks of cancer. Other pathways, such as platinum drug resistance, PI3K-Akt signaling pathway, HIF-1 signaling pathway, and glycolysis are downregulated as well. It has been reported that the aberrant metabolism of cancer cells characterized by high glycolysis facilitates tumor growth and metastasis even in the presence of abundant oxygen. The reprogramming of tumor cell energy metabolism has attracted great interest in terms of its application of a new class of effective anticancer treatment strategies [33, 34]. It will be interesting to examine whether TXX-1-10 regulates cancer metabolism, and further studies will also be conducted to combine TXX-1-10 with other strategies.

Collectively, we developed a novel agent named TXX-1-10, which significantly reduces HPIP expression and regulates HPIP downstream targets. TXX-1-10 has a more effective inhibition on breast cancer cell proliferation, migration, invasion, and metastasis in vitro and in vivo than rimonabant. The gene-expression signature also reflects the tumor-suppressive properties of TXX-1-10 on diverse tumor processes, suggesting HPIP may be an attractive therapeutic target for cancer therapy. Further investigation is warranted to evaluate the efficacy of TXX-1-10 as a clinical candidate drug or in combination with other strategies for cancer treatment.

## Materials and methods

### Chemical synthesis

The chemical structures and synthetic schemes for TXX-1-10 are presented in Supplemental Fig. 2A. Detailed synthetic procedures are provided below.

### Preparation of 5-(4-chlorophenyl)-1-(2,4-dichlorophenyl)-4-methyl-1H-pyrazole-3-formyl chloride (compound b)

Sulfoxide (20 ml) was added into a stirred solution of rimonabant carboxylic acid (3.86 g, 10.12 mmol) in toluene (20 ml). The mixture was stirred at 130 °C for 6 h and evaporated to dryness between 110–130 °C to get the crude product.

### Preparation of 5-(4-chlorophenyl)-1-(2,4-dichlorophenyl)-4-methyl-N-(3-pyridyl-methyl)-1H-pyrazole-3-carboxamide (compound c)

3-aminomethylpyridine (0.64 g, 3.0 mmol) dissolved in dichloromethane (40 ml) was added dropwise to a solution of compound 2 (2.40 g, 3.0 mmol) in dichloromethane (20 ml), and triethylamine (1.20 g, 6.00 mmol) was added to the solution. The mixture was stirred at room temperature overnight. Then the mixture was poured into water (100 ml) and extracted with DCM three times. The organic phase was combined, washed, evaporated, and purified to afford the title compound (1.72 g, yield 60.3%). ^1^H-NMR (400 MHz, DMSO-d6) δ 2.39 (s, 3H), 4.76–4.77 (d, 2H), 7.06–7.42 (m, 9H), 7.66–7.68 (m, 1H), 7.85 (s, 1H), 8.56–8.57 (m, 1H), ESI-MS m/z: 471.2 [M + H]^+^

### Preparation of TXX-1-10

Compound 3 (920 mg, 1.96 mmol) dissolved in N-methylpyrazolidone (8 ml) was added to CH_3_I (840 mg, 5.92 mmol). The mixture was stirred at 35–40 °C for 2 h. The crude product was purified by recrystallization to afford title compound (972 mg, yield 39.1%). ^1^H-NMR (400 MHz, DMSO-d6) δ 2.50 (s,3H), 2.29 (s,3H), 4.36 (s,3H), 7.25–7.27 (m,2H), 7.45–7.47 (m,2H), 7.59–7.62 (dd,1H), 7.77–7.83 (m,2H), 8.04–8.08 (m,1H), 8.08–8.12 (m,1H), 8.68–8.74 (m,1H), 9.54 (s,1H), 11.25 (s,1H), ESI-MS m/z: *m/z*: 473.5 [M + H]^+^

### Cell lines and reagents

All cell lines used in this study were purchased from the American Type Culture Collection (ATCC, Manassas, Virginia, USA). MCF-7, ZR-751, T-47D, MDA-MB-231, MDA-MB-468, MDA-MB-436, A549, H1299, HepG2, SMMC-7721, 786-O, HeLa, PANC-1, BXPC-3, 8305 C, 293 T, LO2, HMEC, and NIH3T3 cells were cultured in DMEM (high glucose) containing 25 mM glucose (Invitrogen), 10% FBS (Hyclone), 1% penicillin (Invitrogen), and 1% streptomycin (Invitrogen). All cell lines were maintained in a humidified incubator at 37 °C and 5% CO_2_. TXX-1-10 and other novel derivatives were designed and synthesized in the laboratory of Professor Zhibin Zheng at National Engineering Research Center for the Emergency Drug through the chemical modification of rimonabant. Compounds were dissolved in DMSO and diluted in normal saline for cell culture studies.

Rimonabant(#T1519L) was purchased from Targetmol, USA; anti-β-actin(#20536-1-AP), anticyclin D1(#26939-1-AP), anti-p21(#10355-1-AP), anti-p27(#25614-1-AP), anti-BAX (#50599-2-Ig), anti-Bcl-2(#12789-1-AP), anti-PARP(#13371-1-AP), anti-Cyclin B1 (#55004-1-AP), anti-HPIP(#12102-1-AP), anti-Ki67(#27309-1-AP), anti-FAK(#12636-1-AP), anti-ERK (#67170-1-Ig), anti-AKT (#10176-2-AP), anti-Paxillin (#22172-1-AP), anti-pS2 (#13734-1-AP), anti-ERα (#21244-1-AP), anti-Vimentin (#10366-1-AP), anti-Slug (#12129-1-AP), anti-Snail (#13099-1-AP), anti-Flag (#66008-3-Ig), anti-HA(#66006-2-Ig), anti-E-Cadherin (#20874-1-AP), anti-N-Cadherin (#22018-1-AP) and anti-Cathepsin D (#55021-1-AP) were purchased from Proteintech; anti-Cleaved Caspase-3(Asp175)(#9661), anti-Caspase 3(#9662), anti-phos-FAK(Y397)(#8556), anti-phos-AKT(T308)(#13038), and anti-CD31(#77699) were purchased from Cell Signaling Technology; anti-phos-Paxillin(S178)(#sc-365020), and anti-phos-ERK (T202/Y204)(#sc-136521) were purchased from Santa Cruz Biotechnology.

### Plasmids and lentiviruses construction

Stable ZR75-1 cell line overexpressing HPIP-copGFP was constructed by inserting PCR-amplified HPIP fragment linked with copGFP tag at the carboxyl terminus into a pCDH plasmid (System Biosciences) using the following primers: HPIP CDS: 5′- GCTCTAGAGCCACCATGGCCTCCTGCCCAGACTCTGATAA-3′ (forward) and 5′-CGGGATCCGCCCCGGTGGTGGTGGTGGTGGCTAT-3′ (reverse); CopGFP CDS: 5′-CGGGATCCATGGAGAGCGACGAGAGCG-3′ (forward) and 5′-ATAAGAATGCGGCCGCGCGAGATCCGGTGGAGCC-3′ (reverse). The 293 T cells were inoculated into six-well plates to 70–90% confluence degree, the DNA-lipid complex was added into the cells according to the manufacturer’s instructions (Invitrogen), and the cells were incubated at 37 °C for 2–4 days to collected lentivirus. Stable cell lines were selected with puromycin 48 h after infection.

### Estimation of brain uptake

Brain uptake clearance was estimated using a sample pooling method according to a previous study [35]. TXX-1-10 and rimonabant, at 5 mM, were injected via the tail vein into male Sprague-Dawley rats (body weight = 200 g ± 15 g). First, a rapid bolus injection was conducted at a flow rate of 150 ml/h for 5 s, followed by a continuous bolus injection at a flow rate of 0.5 ml/h. Venous blood was taken from heparin sodium anticoagulant tubes after administration. The drug concentration in plasma and brain tissue samples were measured by LCMS-8060 liquid chromatography-mass spectrometry (Shimadzu, Japan).

### Cell viability and colony formation assays

For cell viability assay, anchorage-dependent cell viability was evaluated by the CCK-8 Kit (Dojindo Laboratories) according to the manufacturer’s instructions. Cancer cells in complete cell culture medium were seeded in 96-well plates (100 µl per well) at 4000 cells/well to adhere overnight and were then treated. The compounds were prepared in a complete cell culture medium and 100 µl of 2×treatment-containing medium were added to each well for 48 h. Subsequently, 100 µl of cell culture medium containing 10% CCK-8 solution was added to cultured cells, and the mixture was incubated at 37 °C and 5% CO_2_ for 2 h. The OD values were measured at 450 nm using a microplate reader. For colony formation assay, cancer cells treated with TXX-1-10 or vehicle (DMSO) for 48 h were plated in twelve-well dishes in triplicate at 2000 cells per well to grow for 10–14 days. The number of colonies with more than 1.0 mm diameters was scored.

### Cell migration and invasion

For cell migration assay, the confluent monolayers of cells were mechanically scratched using a 200 µl pipette tip. The debris were washed with PBS three times and treated with DMSO or compounds in DMEM (high glucose) without FBS accompanied with mitomycin C (1 μM) treatment. The migrated cells were counted after taking images at 0 and 12 h in the same place. For cell invasion assay, 10 μl liquid Matrigel (BD Biosciences) melted on the ice was added dropwise to the upper surface of the transwell chamber (Corning). Cancer cells were washed with PBS three times and were added to each well with DMSO or compounds treatment-containing medium at 10,000 cells per well accompanied with mitomycin C (1 μM) treatment. After 24 h, 4% paraformaldehyde was used to fix the cells invading the matrix gel membrane, and then the cells were stained with crystal violet. The number of the invaded cells was counted after taking photographs.

### Cell cycle and apoptosis

Cancer cells (1 × 10^6^ cells) were cultured in six-well dishes to adhere overnight and were then treated with compound for 36 h. For cell cycle analysis, cells were fixed in 70% ethanol overnight, washed with PBS three times, and incubated with 500 ml RNase A (0.2 mg/mL) in PBS for 30 min. Propidium Iodide was then added to the cell suspension. Samples were analyzed by a FACS calibur Flow Cytometer (Becton Dickinson). Alternatively, the cells were labeled with propidium iodide and annexin V according to the manufacturer’s instructions (KeyGen Biotech).

### Quantitative reverse-transcription PCR (RT-qPCR)

The samples were homogenized with TRIzol Reagent (Invitrogen), vortexed for 1 min with 200 ml chloroform and centrifuged at 1.2 × 10^4 ^rpm for 15 min at 4 °C. The upper aqueous phase (containing RNA) was precipitated with 400 µl isopropanol at room temperature for 10 min and centrifuged at 1.2 × 10^4 ^rpm for 10 min at 4 °C. The RNA pellets were washed with 70% (v/v) ethanol and then washed with 100% (v/v) ethanol. Then the RNA pellets air-dried and dissolved in 50 µl nuclease-free water. Then 2 mg of total RNA reverse-transcribed to cDNA according to the manufacturer’s recommendation (Takara).

### RNA-Seq

A minimum of 3 μg of total RNA was oligo (dT) selected using the Dynabeads mRNA purification kit (Invitrogen). NEBNext ®UltraTM RNA Library Prep Kit for Illumina ®(NEB, USA) was used to generate a sequencing library. In short, the mRNA was extracted and then purified from total RNA. Double-stranded cDNA was synthesized with these short fragments as templates. The cDNA was end-repaired, ligated to Illumina adapters, size selected on agarose gel (∼250 bp), and PCR amplified. The cDNA library was sequenced on an Illumina HiSeq 6000 sequencing platform (BerryGenomics). The gene-expression levels for each transcript of the exon model were estimated as the number of reads per kilobase per million mapped reads (RPKM) using only uniquely mapped reads in exonic regions. A gene is considered significantly differentially expressed if its expression differs between any two samples with the fold change >2 and the *p* value< 0.05 as calculated by Cufflinks. The RNA-Seq data are available at the Gene Expression Omnibus (http://www.ncbi.nlm.nih.gov/geo/) under accession ID (GSE166371).

### ALDEFLUOR assay

MDA-MB-231 and ZR75-1 cells were collected and suspended in ALDEFLUOR assay buffer. Each sample was treated with 50 mmol/l diethylaminobenzaldehyde (DEAB), a specific ALDH inhibitor, as a negative control. Each sample was treated with containing ALDH substrate (StemCell Technologies) and incubated for 45 min at 37 °C. Cells were analyzed on a flow cytometer (BD Biosciences). FACS data were analyzed with FlowJo software (Treestart).

### Animal models for tumor growth and metastasis

Animal studies were approved by the Institutional Animal Care Committee of Beijing Institute of Biotechnology. Nude mice were purchased from Vital River Laboratory Animal Technology (Beijing) and housed in an SPF animal facility. For tumor xenografts, 5 × 10^6^ MDA-MB-231-Luciferase cells were injected subcutaneously into the axilla of 6-week-old female nude mice. The tumor size was measured at the indicated time using callipers. The tumor volume was estimated according to the following formula: volume = (longest diameter × shortest diameter^2^)/2. When the tumors reached a volume of ∼50 mm^3^, the mice were randomly divided into groups and intraperitoneally injected with rimonabant (30 mg/kg) and TXX-1-10 (30 mg/kg) with an equivalent volume of saline injected in control animals. This experiment was terminated when the maximum tumor size reached ∼1.5 cm in diameter, at which point the tumors were isolated from the animals and weighed. For the metastasis model, 2 × 10^5^ metastasis MDA-MB-231-Luciferase cells were injected into the tail vein of nude mice. The mice were randomly divided into groups and intraperitoneally injected with rimonabant (30 mg/kg) and TXX-1-10 (30 mg/kg) with an equivalent volume of saline injected in control animals. Images of xenograft mice were obtained using a Xenogen IVIS 2000 Luminal Imager once a week.

### Immunohistochemistry

Paraffin sections (3 mm) were mounted on Plus slides and dried in a 60 °C oven. The slides were placed on a Leica BondMax Immunostainer. Antibodies were optimized with a predetermined staining protocol: Ki67, 1:800; CD31 Rabbit mAb 1:500; HPIP Rabbit mAb, 1:500, and cleaved-caspase-3 (Asp175), 1:1000. Slides were dehydrated and cover-slipped with Cytoseal 60 (Richard-Allan Scientific) mounting medium.

### Statistical analysis

All in vitro experiments were performed in triplicate and repeated three times. Differences between variables were assessed by *χ*2 analysis, two-tailed Student’s *t* test, log-rank test. The SPSS software 13.0 or GraphPad PRISM 6 (GraphPad) statistical software package is used to perform all statistical analyses. The data are expressed as the mean ± standard deviation, and *P* < 0.05 was considered statistically significant. In all assays, *p* < 0.05 was considered statistically significant.

## Supplementary information

HPIP-Revised-Supplemental figure legends

The primary screen revealed that molecules derived from rimonabant significantly reduced HPIP expression

The synthesis of TXX-1-10

Physicochemical properties of TXX-1-10 and rimonabant predicted by ADMET Predictor 8.1 program

TXX-1-10 significantly reduces blood-brain barrier (BBB) permeability compared to rimonabant

Evaluation of stemness regulated by TXX-1-10

The effect of TXX-1-10 on HPIP-ERα interaction ERα-targeted genes

Analysis of genes regulated by TXX-1-10

## Data Availability

All data generated or analyzed during this study are included in this published article and its supplementary information files.
