# Supplementary material for: A novel chemical inhibitor suppresses breast cancer cell growth and metastasis through inhibiting HPIP oncoprotein
Source: Cell Death Discov. 2021 Jul 29;7:198. doi: 10.1038/s41420-021-00580-3 (PMC8322322; doi:10.1038/s41420-021-00580-3)
Supplement: Supplementary file 1 — HPIP-Revised-Supplemental figure legends [file 41420_2021_580_MOESM1_ESM.docx]

**Figure S1 The primary screen revealed that molecules derived from rimonabant significantly reduced HPIP expression. A** Summary of the cell viability of ZR75-1 cells stably expressing HPIP-copGFP treated with all compounds (10 μM). The red circle indicates cell viability < 30% treated with the indicated compounds. **B** Chemical structure of rimonabant derivatives inhibiting ZR75-1 cell viability. **C** Representative images of ZR75-1 cells stably expressing HPIP-copGFP treated with the indicated TXX-1-10 observed by a fluorescence microscope, Scale bars: 100 μm. **D** Cell growth inhibitory activity of TXX-1-10 in 24 cell lines was determined by CCK8 cell growth assay. **E** Western blot assay of HPIP expression in the panel of breast cancer cell lines and normal breast cells treated with TXX-1-10 (10 μM) for 24 h. Data shown are mean ± SD of triplicate measurements that have been repeated 3 times with similar results. Statistical significance was assessed by two-tailed Student’s t test. *p < 0.05, **p < 0.01 versus the corresponding control.

**Figure S2 The synthesis of TXX-1-10. A** The synthetic route to TXX-1-10. “a” is a known chemical; “b” represents compound b; “c” represents compound c. **B** ^1^H NMR spectra (400 MHz, chloroform-*d*, 298 K) of **c**. **C** Electrospray ionization mass spectrum of **c**. **D** ^1^H NMR spectra (400 MHz, chloroform-*d*, 298 K) of **TXX-1-10**. **E** Electrospray ionization mass spectrum of **TXX-1-10**.

**Figure S3** **Physico-chemical properties of TXX-1-10 and rimonabant predicted by ADMET Predictor 8.1 program**. **A** LogBB, S+logP, S+logD of TXX-1-10 and rimonabant. **B** The toxicity and physiochemical properties of TXX-1-10 and rimonabant.

**Figure S4 TXX-1-10 significantly reduces blood-brain barrier (BBB) permeability compared to rimonabant. A** Representative image of TXX-1-10 distribution in plasma (top) and brain tissue (bottom) measured by LCMS-8060 liquid chromatography-mass spectrometry. **B** Representative image of Rimonabant distribution in plasma (top) and brain tissue (bottom) measured by LCMS-8060 liquid chromatography-mass spectrometry. **C** Quantification of distribution of TXX-1-10 (n = 4) and rimonabant (n = 3) in in paired plasma and brain tissue in the same rat measured by LCMS-8060 liquid chromatography-mass spectrometry. **D** Histograms show the relative level TXX-1-10 (n = 4) and rimonabant in plasma and brain tissue (n = 3). Statistical significance was assessed by two-tailed Student’s t test. *p < 0.05, **p < 0.01 versus the corresponding control.

**Figure S5 Evaluation of stemness regulated by TXX-1-10. A** The cell stemness of ZR75-1 cells treated with TXX-1-10, Rimonabant, or vehicle for 48 h. Cell stemness was evaluated by flow cytometry after staining with ALDEFLUOR^TM^ Reagent for 30 min at 37°C. The stained corresponding diethylaminobenzaldehyde (DEAB) is used to control for background fluorescence (upper). Histograms show the relative positive stemness cell (bottom). **B** The cell stemness of MDA-MB-231 cells treated with TXX-1-10, Rimonabant, or vehicle for 48 h. Cell stemness was evaluated by flow cytometry after staining with ALDEFLUOR^TM^ Reagent for 30 min at 37°C. The stained corresponding diethylaminobenzaldehyde (DEAB) is used to control for background fluorescence (upper). Histograms show the relative positive stemness cell (bottom).Data shown are mean ± SD of triplicate measurements that have been repeated 3 times with similar results. Statistical significance was assessed by two-tailed Student’s t test. **p* < 0.05, ***p* < 0.01 versus the corresponding control.

**Figure S6 The effect of TXX-1-10 on HPIP-ERα interaction ERα targeted genes. A** Interaction of overexpressed HPIP with overexpressed HPIP or ERα in mammalian cells. MCF-7 cells were transfected with expression plasmids as indicated. Immunoprecipitation (IP) was performed using anti-Flag monoclonal antibody, and immunoblotted (IB) with anti-Flag and anti-HA monoclonal antibody. **B** Western blot analysis of ZR75-1 with TXX-1-10 (10 μM) treatment for 24 h or HPIP overexpression.

**Figure S7 Analysis of genes regulated by TXX-1-10. A-B** A volcano plot illustrating differentially regulated gene expression from RNA-seq analysis between the control Vehicle and TXX-1-10 treatment (C) and rimonabant treatment (D), respectively. Genes upregulated and downregulated are shown in red and green, respectively. Values are presented as the log2 of tag counts. Overlay plots of regulated mRNA expression of MDA-MB-231 cells treated with TXX-1-10 (TXX) and rimonabant (SR) treatment. **D-H** qRT-PCR validation analysis shows the mRNA expression fold change of DNA replication(D), cell cycle (E), apoptosis (F), cell adhesion(G) and cell migration/invasion/metastasis (H) associated genes with TXX-1-10, rimonabant or vehicle control treatment in ZR75-1 cells. Data shown are mean ± SD of triplicate measurements that have been repeated 3 times with similar results. Statistical significance was assessed by two-tailed Student’s t test. *p < 0.05, **p < 0.01 versus the corresponding control.
