# Supplementary figures and images for: A novel chemical inhibitor suppresses breast cancer cell growth and metastasis through inhibiting HPIP oncoprotein
Source: Cell Death Discov. 2021 Jul 29;7:198. doi: 10.1038/s41420-021-00580-3 (PMC8322322; doi:10.1038/s41420-021-00580-3)

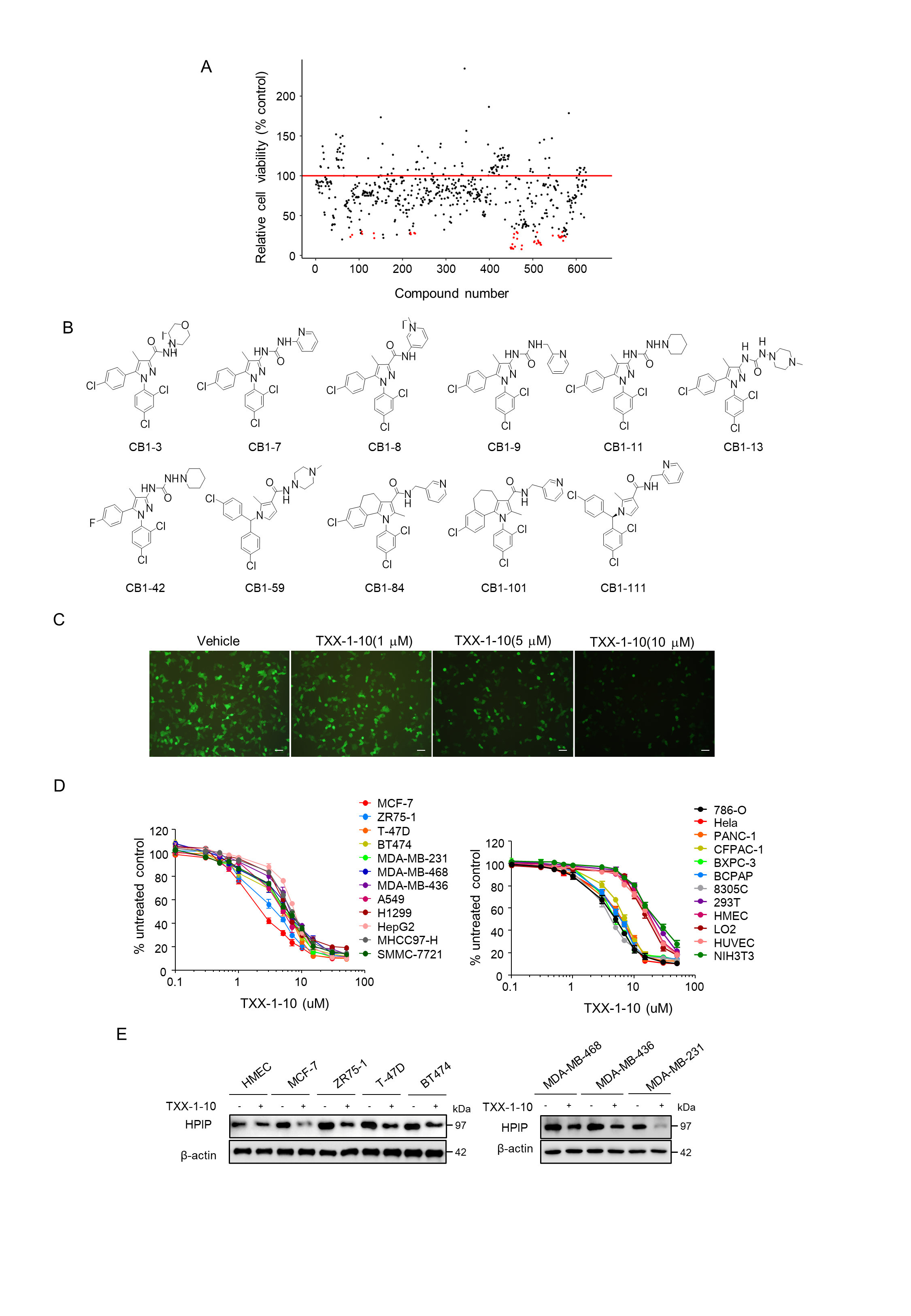

Supplement: Supplementary file 2 — The primary screen revealed that molecules derived from rimonabant significantly reduced HPIP expression [file 41420_2021_580_MOESM2_ESM.tif]

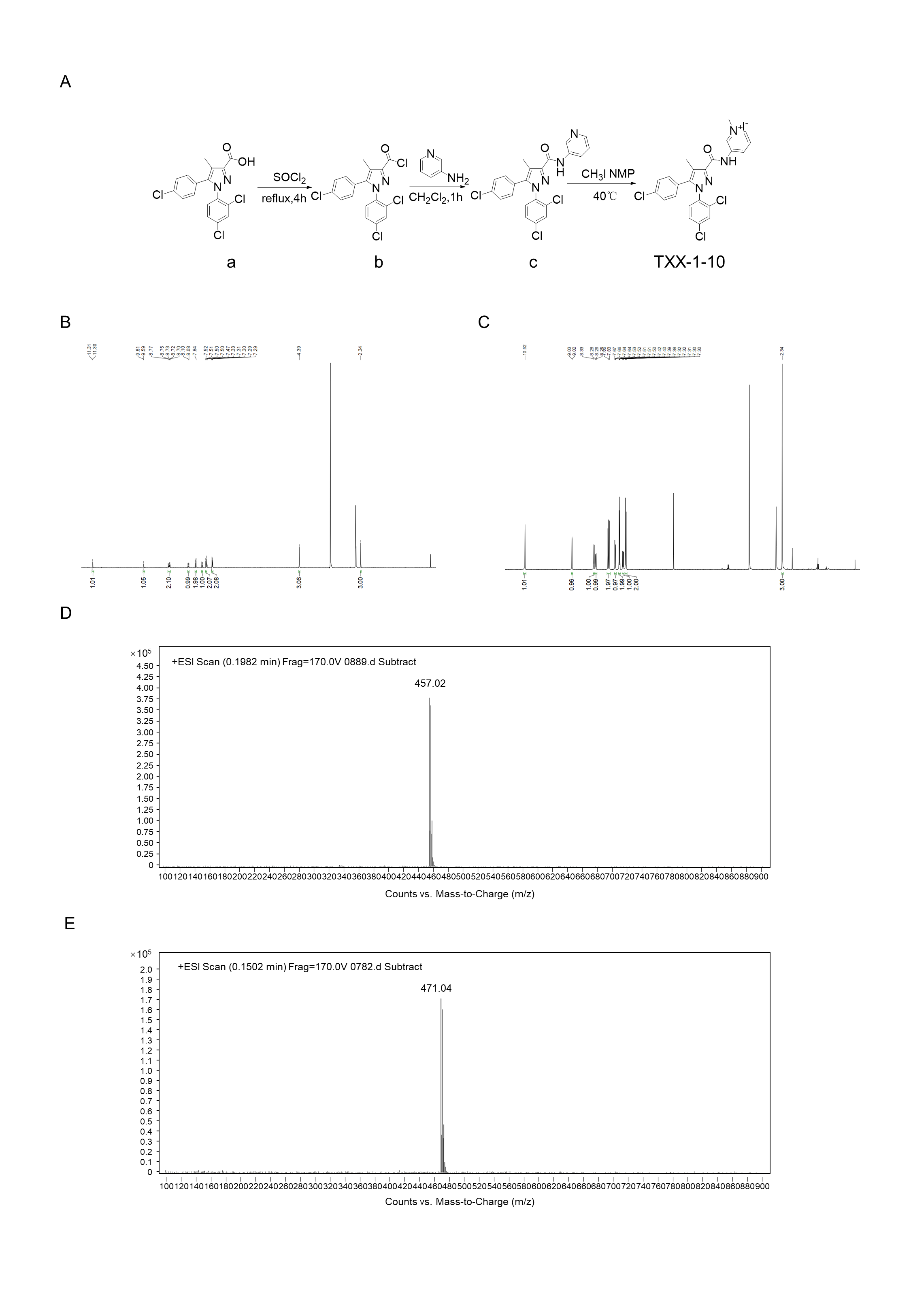

Supplement: Supplementary file 3 — The synthesis of TXX-1-10 [file 41420_2021_580_MOESM3_ESM.tif]

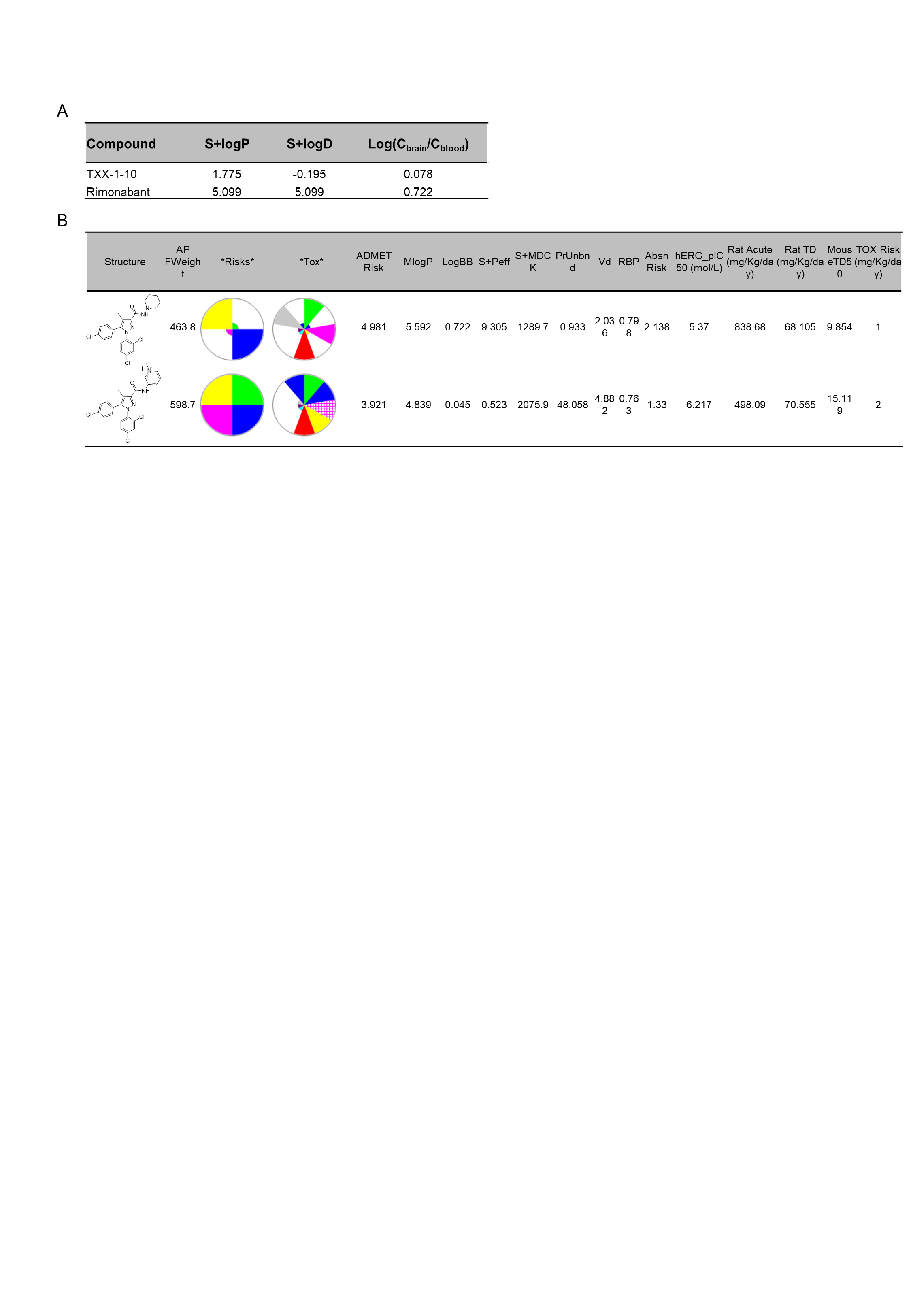

Supplement: Supplementary file 4 — Physicochemical properties of TXX-1-10 and rimonabant predicted by ADMET Predictor 8.1 program [file 41420_2021_580_MOESM4_ESM.tif]

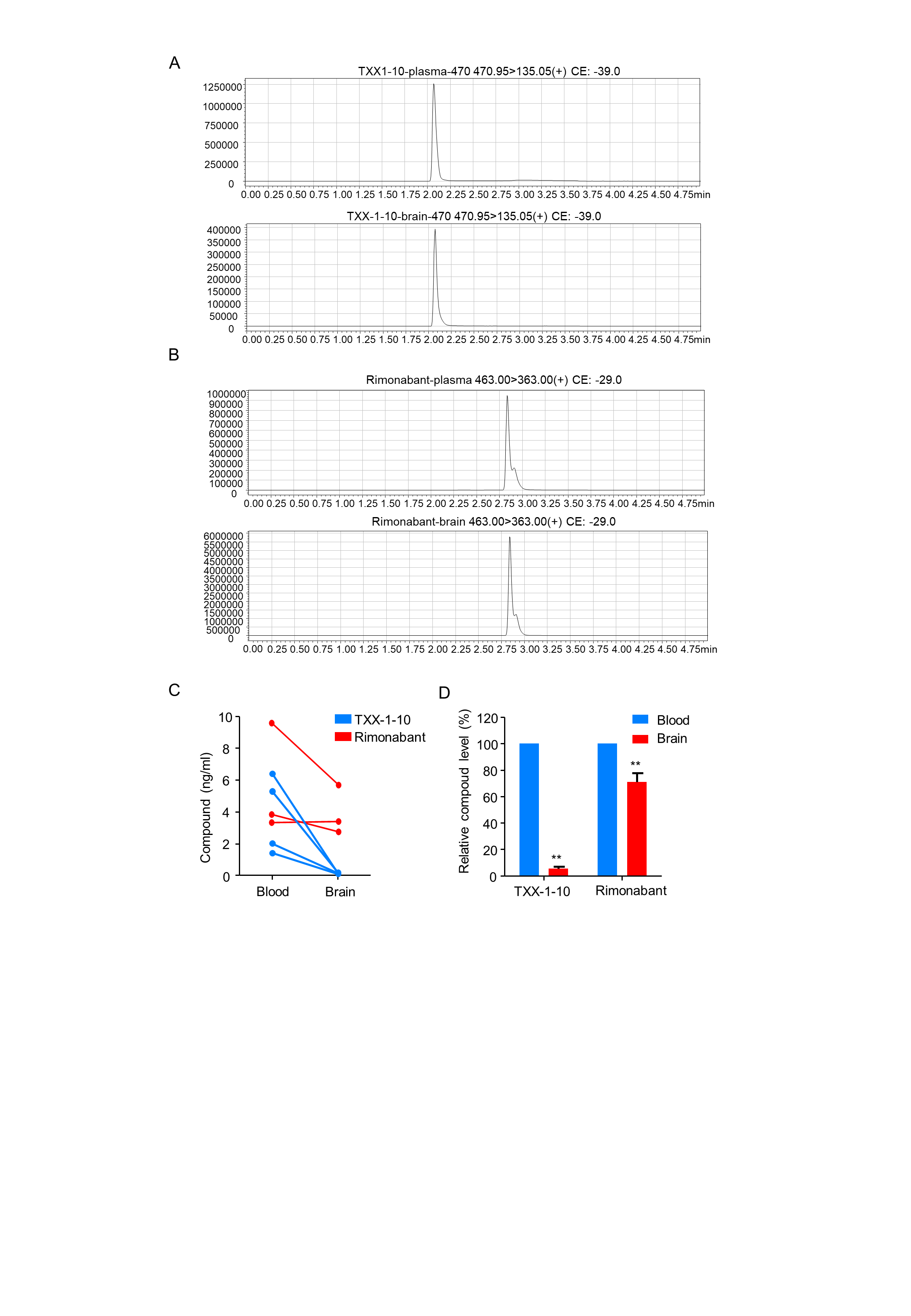

Supplement: Supplementary file 5 — TXX-1-10 significantly reduces blood-brain barrier (BBB) permeability compared to rimonabant [file 41420_2021_580_MOESM5_ESM.tif]

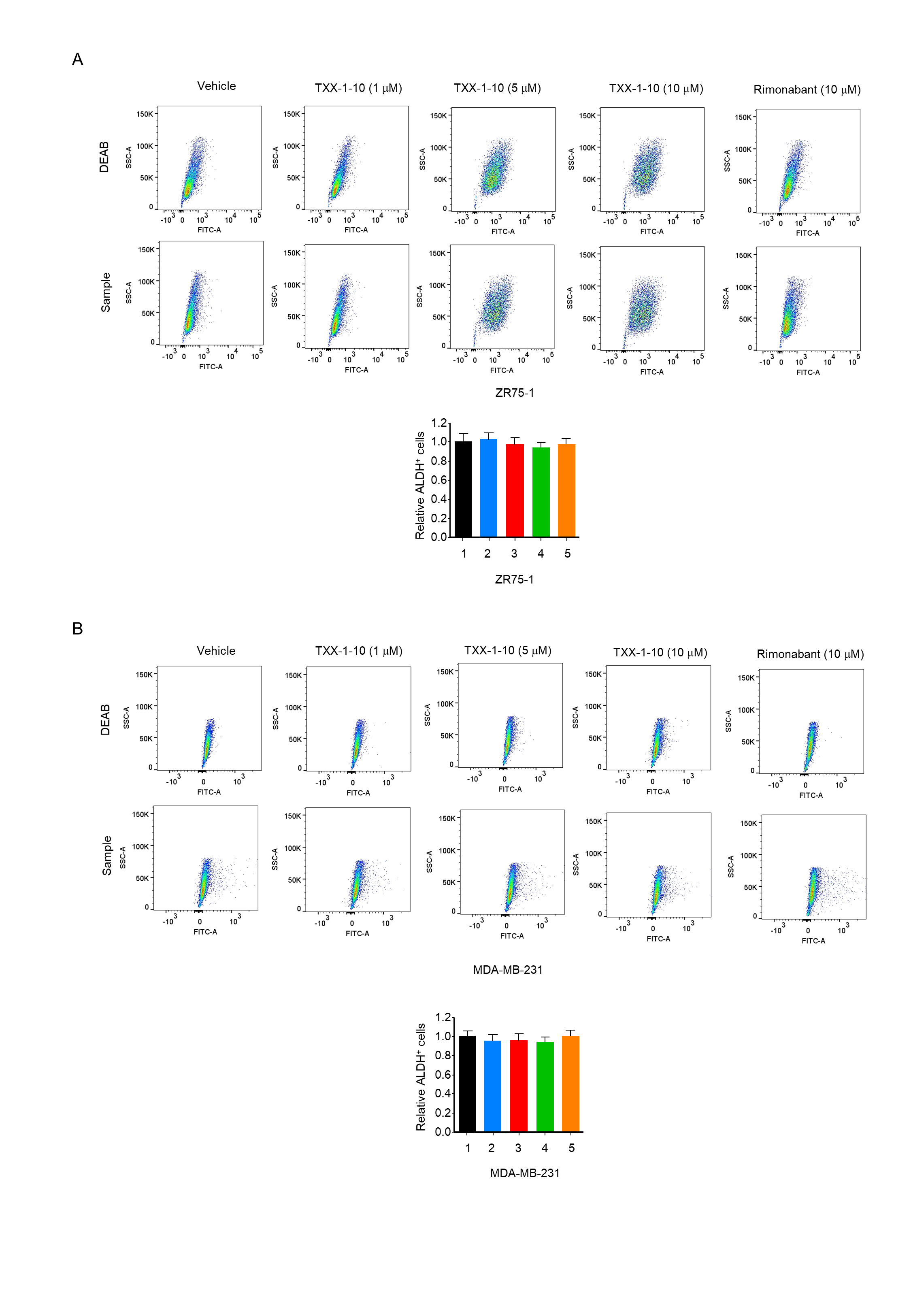

Supplement: Supplementary file 6 — Evaluation of stemness regulated by TXX-1-10 [file 41420_2021_580_MOESM6_ESM.tif]

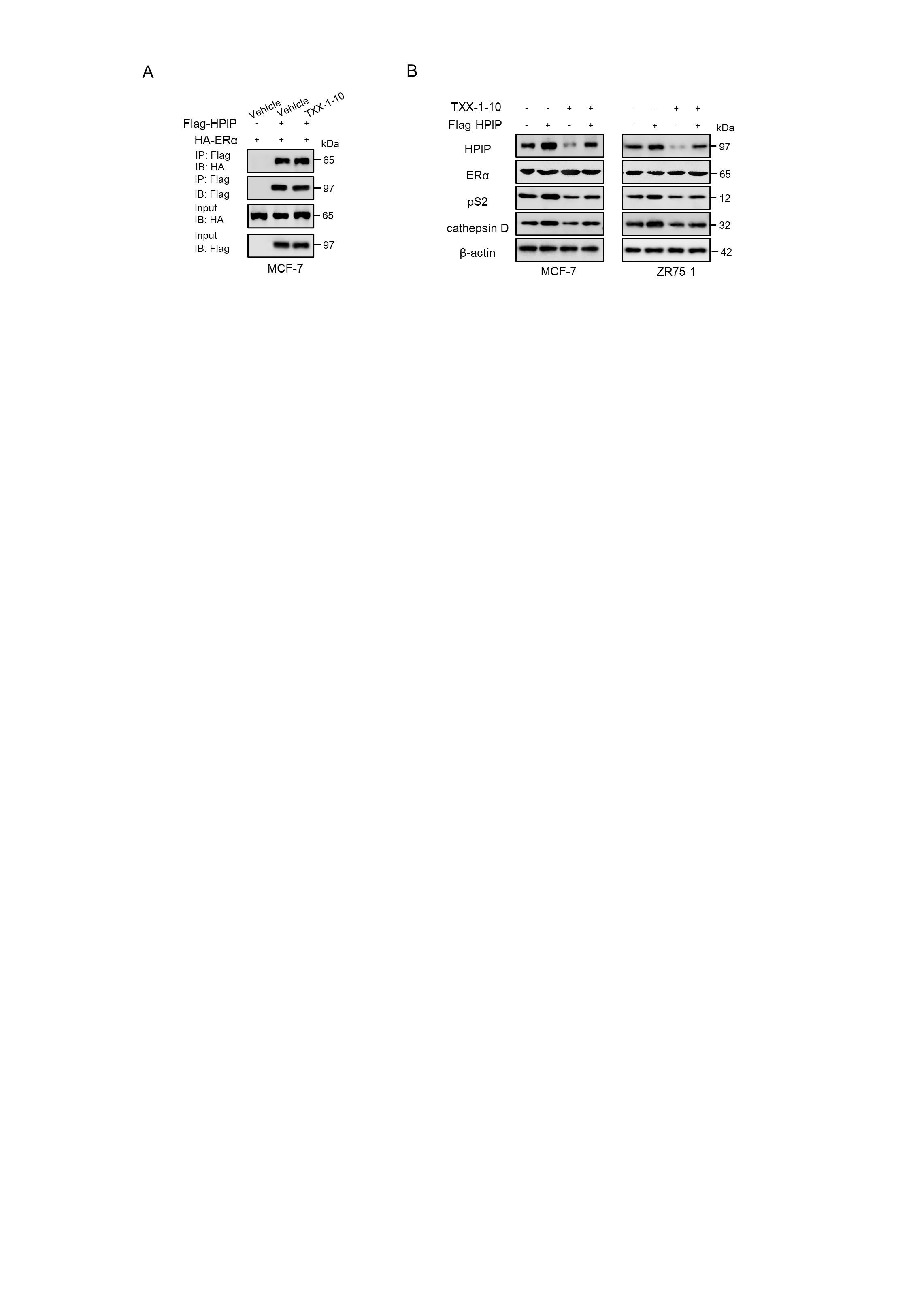

Supplement: Supplementary file 7 — The effect of TXX-1-10 on HPIP-ERα interaction ERα-targeted genes [file 41420_2021_580_MOESM7_ESM.tif]

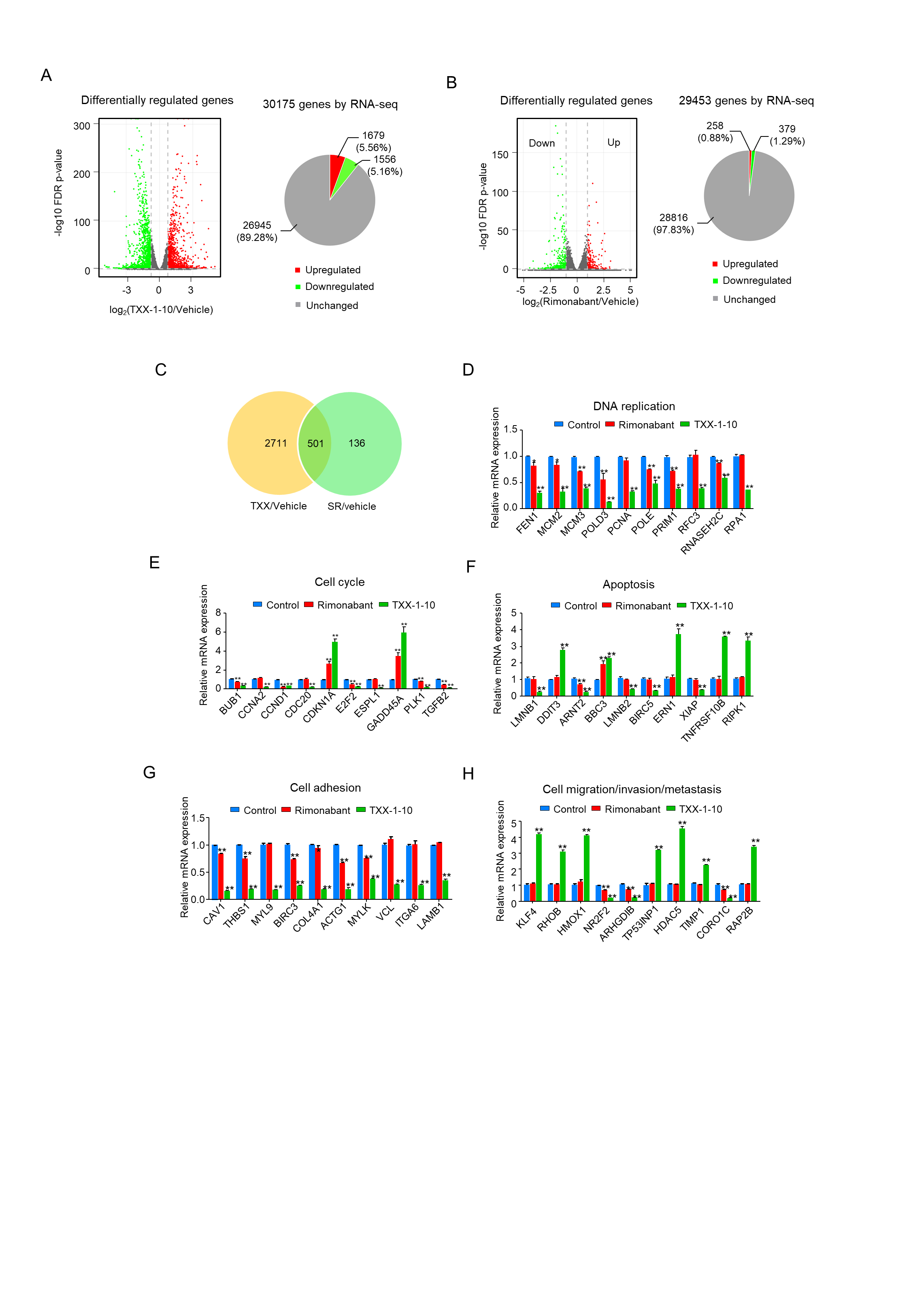

Supplement: Supplementary file 8 — Analysis of genes regulated by TXX-1-10 [file 41420_2021_580_MOESM8_ESM.tif]
